# Ambivalence and the biopolitics of HIV pre-exposure prophylaxis (PrEP) implementation

**DOI:** 10.1057/s41285-020-00154-w

**Published:** 2021-01-14

**Authors:** Mark Gaspar, Travis Salway, Daniel Grace

**Affiliations:** 1 Dalla Lana School of Public Health, University of Toronto, 155 College Street, Room 510, Toronto, ON M5T 3M7 Canada; 2 Faculty of Health Sciences, Simon Fraser University, Blusson Hall, Room 11300, 8888 University Drive, Burnaby, BC V5A 1S6 Canada; 3 Dalla Lana School of Public Health, University of Toronto, 155 College Street, Room 556, Toronto, ON M5T 3M7 Canada

**Keywords:** Ambivalence, Biopolitics, HIV, PrEP, Gay, bisexual, queer and other men who have sex with men, Epistemology

## Abstract

Ambivalence, the vacillation between conflicting feelings and thoughts, is a key characteristic of scientific knowledge production and emergent biomedical technology. Drawing from sociological theory on ambivalence, we have examined three areas of debate surrounding the early implementation of HIV pre-exposure prophylaxis, or PrEP, for gay, bisexual, queer, and other men who have sex with men in Canada, including epistemology and praxis, clinical and epidemiological implications, and sexual politics. These debates are not focused on the science or efficacy of PrEP to prevent HIV, but rather represent contradictory feelings and opinions about the biopolitics of PrEP and health inequities. Emphasizing how scientists and health practitioners may feel conflicted about the biopolitics of novel biomedical technologies opens up opportunities to consider how a scientific field is or is not adequately advancing issues of equity. Scientists ignoring their ambivalence over the state of their research field may be deemed necessary to achieve a specific implementation goal, but this emotion management work can lead to alienation. We argue that recognizing the emotional dimensions of doing HIV research is not a distraction from “real” science, but can instead be a reflexive site to develop pertinent lines of inquiry better suited at addressing health inequities.

## Introduction

We begin by drawing on an anecdote as a non-representative “research device” with the capacity to prompt reflexivity on the current state of knowledge (Race 2016). In November 2017, we attended the Community-Based Research Centre’s annual Summit in Vancouver, British Columbia. The Summit is one of most important community-led meetings on the health of gay, bisexual, queer, and other men who have sex with men (GBM) in Canada. The Summit’s theme that year was “Romancing the Package: Optimizing Combination Prevention”. The topic was timely. In 2015, a clinical trial confirmed that people living with HIV, including GBM, achieving an undetectable viral load, usually because of antiretroviral treatment, cannot transmit the virus (Rodger et al. 2016). This led to the Canadian HIV sector’s enthusiastic adoption of the “U = U” or “Undetectable Equals Untransmittable” education campaign (Gaspar 2019; Grace et al. 2020) and has added to the discourse of “Treatment as Prevention” (TasP), a population-oriented strategy aimed at reducing transmission through the early initiation of HIV antiretrovirals by recent seroconverts (Lloyd 2017). Then in 2016, Health Canada approved HIV antiretrovirals as pre-exposure prophylaxis therapy (PrEP)—the regular use of HIV medications by HIV-negative persons to prevent HIV infection (Morgan et al. 2018).

While attendees at the 2017 Summit discussed a host of pertinent issues—including substance use, other sexually transmitted infections (STIs), and mental health—PrEP was undeniably its headliner. The keynote speaker enthusiastically discussed how PrEP helped to reduce HIV rates by 40% among gay men in London, England (CBRC 2017). Another panel on increasing PrEP access in Canada received standing ovations and thunderous applause (CBRC 2018). The benefits of PrEP were clearly communicated: declining HIV incidence and less HIV stigma and anxiety. Our job now as a community of scientists and health practitioners was to figure out how to “sell” the benefits of antiretrovirals to GBM—we needed to “romance the package” as it were, making pharmaceuticals and frequent HIV and STI testing not just available, but *desirable*. Indeed, PrEP has since become a foundational pillar of the HIV response in Canada. There has been a keen interest in better understanding the barriers GBM face to accessing it (Grace et al. 2018; Newman et al. 2018) and why PrEP uptake has been slow among “high risk” GBM (Knight et al. 2016). Medical experts have expressed that there is no reason why sexually active GBM should not be on PrEP (CBRC 2018).

Given that GBM currently represent an estimated 52.5% of incident HIV cases in Canada (Public Health Agency of Canada 2018), we were not surprised that PrEP played an important role at the Summit. Indeed, we would have been worried if the field was not discussing PrEP. All of us are directly involved in research projects interested in expanding PrEP access. However, something about how the conversation on PrEP was structured left us *ambivalent*—vacillating between accepting and even celebrating PrEP on the one hand, and being deeply concerned over what avid attention to PrEP may be occluding or even perpetuating, on the other. Our reactions echo Auerbach’s and Hoppe’s (2015) observations that PrEP discourse is often organized by extreme contradictions: “either PrEP holds the promise to ending the HIV pandemic or PrEP is an insidious strategy that will exacerbate HIV epidemics and attendant social ills” (p. 2).

Since the Summit, our experiences at multiple HIV forums across Canada have made it clear that many scholars and activists are also trying to reconcile with PrEP ambivalence. For example, McClelland (2019) writes

Having HIV means living with a sort of cognitive dissonance—holding multiple contradictory ideas in one’s head at once. […]. Yes, it can feel counterintuitive to be advocating for a preventative pill for HIV-negative people when there are still over 10 million people globally living with the virus without access to anti-HIV drugs. Yes, that pill is now being primarily marketed to wealthy gay men in the Global North, including the settler-colonial state of Canada. It seems the epitome of globalized capitalism, patriarchy, white supremacy and medical apartheid wrapped up in one not-so-tiny capsule.

Indeed, PrEP being framed as a panacea—something that GBM have advocated for and ostensibly *need* to be healthy—has generated a strong defensiveness over its use. As McClelland notes:

The barrage of marketing and hype means there has been little room for conversation or dissent about what PrEP means, let alone the decision to take it. The drug has been framed as a polemic: either you are against it, meaning you are sex-negative, slut-shaming and against gay male liberation, or you aren’t.

In this paper, we demonstrate how critical attention to this ambivalence can foster productive dialogue on the biopolitics of PrEP and health inequities. We begin with a review of the sociological theory on ambivalence before charting out key areas of debate on PrEP.

### Ambivalence, science, and biopolitics: embodied subjects and feelings about thinking

Though ambivalence is often colloquially understood as simply indifference or indecisiveness, it is more accurately defined as the “oscillation or tension between opposite poles of feeling and thinking” (Marent et al. 2018, p. 134). Ambivalence is not a specific emotion or thought process, but rather a state marked by (potentially painful) conflict between contrasting feelings and rationales. It emerged conceptually from psychoanalysis to refer to situations where a person simultaneously experiences oppositional emotions (i.e. love/hate) towards the same object (Gould 2009). Freud argued that ambivalence is typically resolved by an individual gravitating towards one emotional state over the other.

Ambivalence has since been adopted by sociologists as a “middle-order” concept to explain the “‘countervailing forces’ of social structures in which paradox and contradiction arise from situated practices” (Arribas-Ayllon and Barlett 2013, p. 337). Sociologically, ambivalence stems from the tensions between norms and counter-norms in a social environment (Hillcoat-Nallétamby and Phillips 2011). These norms may not operate dialectically; contradictions may never be resolved. As such, ambivalence can be understood as “a normal rather than a pathological state of societies”, one which is highly characteristic of modern sociotechnical change and scientific risk assessment (Marent et al. 2018, p. 134).

Critical attention to ambivalence connects to a sociological literature on emotions that conceives of social actors as embodied, emotional subjects and understands that all decision-making is shaped by emotional experience as much as “rational” abstraction or cognitive processes (Freund 1990). This includes scientific knowledge production. Medical sociologists have often reified the primacy of reason over emotion associated with Western epistemology and biomedicine (Freund 1990). While social scientists may attend to the emotional experiences of lay actors, less frequently do they reflexively address how emotions influence *their own* decision-making. Yet, scientists also feel about what they think. Consequently, science can be heavily structured by ambivalence based on competing values and political commitments (Arribas-Ayllon and Barlett 2013). For example, scientists often face normative tensions between autonomy versus collaboration, competition versus cooperation, quality versus quantity, and intrinsic versus instrumental value (Arribas-Ayllon and Barlett 2013).

Ambivalence has also been noted as a fundamental characteristic of emergent biomedical technology (Marent et al. 2018). For instance, Persson (2004) has drawn on Derrida’s conceptualization of the *pharmakon* (the Greek term for drug) as simultaneously *both cure and poison*, to investigate the dual capacities of early antiretroviral regimes to improve the health and longevity of people living with HIV *and* cause significant bodily disfigurement. This conflicting duality leads Persson to contend that antiretrovirals are inherently saturated with ambiguity and ambivalence. Persson stresses that ambivalence towards emergent pharmaceutical technologies is not reducible to their effects on the physical body, but is also shaped by contrasting “cultural ideas about self and body, about illness and health, efficacy and responsibility” (p. 46).

Gould (2009) has used ambivalence conceptually to explicate the early dynamics of AIDS activism, demonstrating how gay and lesbian communities had to manage ambivalent feelings towards heteronormative society, simultaneously reconciling a desire for inclusion with anger towards dominant society’s negligence. Drawing on Bourdieu, Gould argues that an *emotional habitus* structures the reconciliation of ambivalence in a field, making some emotions and associated plans of action expressible, while suppressing others. During the early AIDS crises, the reigning emotional habitus was structured by shame, leading gay and lesbian communities to be more cooperative with the state. However, by the late 1980s the emotional habitus shifted to one of pride and then anger, which encouraged more militant activism. The resolution of ambivalence towards anger played a role in biopolitically framing the lives of sexual minorities in the context of the AIDS crisis as worthy.

Biopolitics here refers to processes of power that simultaneously discipline at the individual level and regulate at the population level to optimize some life (to make live) while disallowing other life (to let die) (Foucault 1978/1990). Biopolitical forces determine which lives matter (which lives we invest in) and how specific investments in life come to regulate bodies and subjectivity (Lloyd 2017). Emergent pharmaceutical technologies, especially ones with potentially enormous consequences on population health outcomes and quality of life, bring to the surface questions about how they can either strengthen or alleviate underlying biopolitical forces that “make live” or “let die”. Understanding the biopolitics of novel drugs requires, then, investigating the social norms and counter-norms that shape reactions to a new pharmaceutical intervention. And where there are norms there is ambivalence.

Below we examine three interrelated areas of debate on PrEP implementation: epistemology and praxis, clinical and epidemiological implications, and sexual politics. We focus on how these debates reveal ambivalent tensions regarding the biopolitics of PrEP. We then conclude by addressing how this ambivalence can affect HIV social scientists and health practitioners as embodied, emotional subjects.

## Epistemology and Praxis

The advent of PrEP has resurfaced long-standing epistemological tensions in the HIV field between biomedical and socio-structurally oriented research, and the relationship between knowledge production and action (Adam 2011; Mykhalovskiy and Rosengarten 2009; Roberts and Matthews 2012). A sub-field of interdisciplinary theoretical and empirical scholarship, often termed “critical social science in HIV” (Mykhalovskiy and Namaste 2019), emerged at the start of the HIV epidemic to offer counter narratives to dominant biomedical approaches and to incorporate understandings of power/inequality, sexuality/pleasure, culture, and critical epistemology to the global public health response (Mykhalovskiy and Rosengarten 2009). These critical scholars have noted that in order to be effective, biomedical interventions need to take into consideration the interplay between social, cultural, political, and economic forces that fundamentally determine health (Kippax and Stephenson 2012; Kippax et al. 2013). Nonetheless, since the arrival of effective antiretroviral regimes in 1996, the promotion of biomedical interventions by public health authorities as *the* way to address the HIV epidemic has turned socio-structural concerns into ancillary matters, ones to be considered insofar as they limit the adoption of effective biomedical treatments rather than being necessary objects of analysis and intervention in their own right (Kippax and Stephenson 2012; Mykhalovskiy and Rosengarten 2009). Consequently, scholars have repeatedly noted the reduced role and influence of critical social science and humanities perspectives in the HIV field in Canada and globally (Mykhalovskiy and Rosengarten 2009; Mykhalovskiy and Namaste 2019). Nguyen et al. (2011) term this process *biomedical triumphalism*, a set of discursive strategies that can evacuate the social justice underpinnings of the HIV epidemic in service of biomedically dominant narratives of success. Given the historical centrality of community mobilization efforts to combat HIV globally, the rise of biomedically triumphant narratives have been described as disconcerting (Kippax et al. 2013; Mahajan 2017).

Biomedical triumphalism can frame knowledge production on structural factors as inherently “non-scientific”, less actionable and as barriers to the “real” work of “getting drugs into bodies” (Nguyen et al. 2011). The rise of evidence-based medicine (Mykhalovskiy and Rosengarten 2009), with its emphasis on randomized control trials and quantitative metrics for programme evaluation, has made it so that scholarly interest in the structural components of the epidemic can be dismissed as inappropriate distractions (Mahajan 2017, p. 165). Indeed, the effectiveness of structural interventions (e.g. changes in gender norms, income redistribution programmes) at improving HIV incidence is more difficult to prove than the impact of proper adherence to a drug plan (Roberts and Matthews 2012).

For example, Gaspar (2019) has shown how the fields of GBM health and HIV in Canada were deeply invested for over a decade in resolving the “treatment optimism” problem, the epidemiological hypothesis that increasing HIV incidence among GBM could be explained by their growing reliance on undetectable viral load as a prevention strategy. This theory proved to be inaccurate on biological grounds once undetectability’s prevention benefits were confirmed. Nonetheless, Gaspar avers that even before “U = U” messaging this epidemiological narrative was already divorced from the lived experiences of HIV-negative men, many of whom were quite sceptical of or unknowledgeable about undetectable viral load. Recent research by Grace et al. (2020) has confirmed the existence of “untransmittable skepticism” among some GBM in Canada despite “U = U” messaging. They argue that while some GBM understand that undetectability signifies *low risk*, GBM remain hesitant to equate it with *no* risk of infection and, as such, are uncertain about how exactly to incorporate “U = U” into their sexual decision-making.

Nonetheless, as Gaspar (2019) argues, the problem with the “treatment optimum” hypothesis was not merely that a biomedical theory proved to be inaccurate, but rather, by epistemologically centring biomedical technology as the dominant entry point for understanding GBM’s health and sexuality, the field often failed to address many structural issues (for example, poor HIV testing services, confusing immigration policy, ambiguous and conflicting prevention messaging) that put these men at risk or that produced HIV stigma and unnecessary anxiety. Nonetheless, “treatment optimism” reigned as a leading hypothesis organizing knowledge production in the field because it was a convenient metric, easy to collect in surveys and thus favourable to sustaining high research productivity (Gaspar 2019). The failure of the “treatment optimism” hypothesis, however, demonstrates the need to be critical of what centring biomedical decision-making as *the* frame for understanding GBM’s health needs and sexuality may be occluding.

Indeed, biomedical triumphalism can be noted by the fact that many published empirical social scientific papers about PrEP are often products of *sub*-studies of larger biomedical studies. These epistemological relationships arguably limit what can be said (politically) about PrEP. Under a biomedically dominated field, a concern with the “social” is narrowed down to understanding how individuals adhere to new treatment or prevention plans (Dowsett 2017; Roberts and Matthew 2012). Kippax and Stephenson (2012) argue that scientific concerns around PrEP are reduced to “implementation challenges” regarding testing and obedience to a daily pill regime. Another key implementation concern is understanding why some “high risk” GBM are reluctant to take PrEP (Knight et al. 2016). In a biomedically triumphant field where PrEP is *the* answer (along with TasP) to a public health crisis, those who refuse to opt into a PrEP regime are framed as problems to be solved. This avid interest in understanding the reluctance of some “high risk” GBM to get on PrEP mirrors the HIV field’s long history of generating fashionable behavioural theories (i.e. AIDS optimism, treatment optimism, condom fatigue) to explain why some GBM “failed” to wear condoms—theories that were quite often unable to account for the actual lived experience of GBM (Adam 2011; Dowsett 2009).

Thus, we are more likely to hear about research exploring whether GBM find PrEP to be a compelling option or if they have been recommended PrEP by a service provider, than work discussing how public policies underfund local sexual health clinics and healthcare more generally in Canada. Often absent in GBM scientific communities are discussions on neoliberal austerity, weakened social assistance programmes, student debt, unaffordable housing, an unlivable minimum wage, and the rise in precarious (under-paid) contract work. While such dynamics are rarely given focal attention at national forums on HIV and GBM health, they do come up as serious concerns in the context of research where GBM are not just consigned to discussing their sexual behaviour (Gaspar et al. 2019b).

One structural debate that has emerged in Canada (and globally) is the price of PrEP. Without insurance, PrEP can cost around $250 CAD a month. While PrEP is still not covered for all age groups in some Canadian provinces (like Ontario), community advocates have successfully mobilized to get PrEP onto many provincial registries. Nonetheless, advocating for PrEP for a specific group of men misses the opportunity to address the lack of a national health insurance programme in Canada, the exploitative link between health insurance with secured employment, and the broader inaccessibility of numerous life-saving/extending treatments and healthcare for many marginalized people living in Canada (Martin 2018). Mass job loss associated with the current COVID-19 pandemic has only further highlighted how linking pharmaceutical access with employment-based insurance benefits (which is how most GBM can afford PrEP in Ontario) remains disastrous public health policy, and ripe for sharpening HIV class-based inequities. Moreover, a myopic attention to PrEP access diminishes real concerns about the commodification/corporatization of healthcare more broadly (Young et al. 2015). By focussing strictly on advocating for PrEP access for GBM, the social-political environment that sustains inaccessibility to healthcare in Canada remains unquestioned. Framing PrEP’s cost as an implementation barrier to be instrumentally rectified arguably excuses, and possibly even replicates, the very systems and neoliberal ideologies which implemented these barriers in the first place.

Thus, for critical social scientists who have been arguing for the necessity of attending to the social and structural dimensions of HIV, the advent of PrEP can produce ambivalent reactions. On the one hand, this tool can dramatically improve the quality of life and health outcomes of populations at risk for HIV. Given that this has been the central aim for all us working in the field, an official commitment to PrEP implementation is a *significant* victory. On the other hand, this biomedical victory continues to threaten the necessity of considering creative social and structural solutions to improve health inequities for GBM in Canada (and beyond). Hence, this is why experiences like the one we had at the 2017 Summit that celebrated “romancing the biomedical package” can provoke a strong sense of discomfort over what norms PrEP implementation science is reifying. One is simultaneously caught celebrating the newfound potentials of PrEP, while also feeling disheartened at the flattening out of any discursive and political attention to the socio-structural components of the epidemic. Moreover, our ambivalence at the Summit arguably stemmed from a recognition of our complicity within the very epistemological and biopolitical systems we critique. Though, as social scientists, we are critical of PrEP triumphalism, we also hold active positions within the scientific and community networks advancing this biomedical narrative, and benefit professionally from the field’s focus on PrEP. This contradiction is perhaps inherent to much HIV critical social science (Mykhalovskiy and Namaste 2019). Critical social scientists are regularly embedded in the same institutions that they are otherwise critical of, and both contribute to and benefit from biomedical triumphalism in their research. These contradictions can produce a discomforting ambivalence when one is sitting in a room bursting with applause over pharmaceutical advances with no mention of the structural forces marginalizing the very populations who will ostensibly be aided by such advances.

## Clinical and epidemiological consequences

Questions have also been raised as to whether PrEP may be reinforcing health inequities. Scholars have noted a paradox whereby the “ideal PrEP user” is also the “fallible man”, someone simultaneously “vulnerable” to HIV acquisition by not having followed previous prevention guidelines but suddenly capable of complying with a complex prevention programme (Holt 2015; Trachman and Girard 2018). Individuals most at risk for HIV (such as those with serious mental health or substance use issues, insecure housing) may actually be deemed “too high risk” to adequately adhere to PrEP, while those with lower risk sexual practices for HIV may be more likely to be deemed eligible because they will be “good” (adherent) PrEP users (Auerbach and Hoppe 2015). Moreover, given that PrEP access is often linked to employment and/or private insurance, PrEP’s implementation will likely aggravate existing health inequities related to socioeconomic status (Morgan et al. 2018). These contradictions can lead to ambivalence among health practitioners who have to reconcile that *access* to PrEP is not the same as the *need* for PrEP.

Race is another factor that can significantly limit accessibility (Calabrese et al. 2014). Some have argued that the PrEP clinical guidelines in Canada omit important considerations that contribute to HIV risk among Black men and women, and thus will continue to foster health inequities in HIV by under-prescribing PrEP to Black communities (Nelson et al. 2019). PrEP implementation in Canada was focussed primarily on increasing access for GBM first (including to the exclusion of access for women). Early work to get PrEP on the provincial registry in British Columbia ignored how Indigenous GBM and two-spirit folk already had free access to PrEP, especially egregious given that Indigenous communities experience some of the highest HIV incidence rates in the country (Public Health Agency of Canada 2018; Stillwagon and Pruden 2019). Thus, the implementation of PrEP for GBM in Canada has reified debates on differences between healthcare access for GBM of colour and white GBM, and between Indigenous and settler GBM. From this view, an ambivalence to PrEP triumphalism cannot be solved simply by expanding PrEP access, but requires acknowledging the historical role of white and settler privilege, and racism and colonization within the GBM and HIV healthcare sectors.

Conversely, there are concerns with potentially “over-prescribing” PrEP. Some have been critical of PrEP use as essentially an anti-anxiety medication for HIV- and sex-related fears, particularly among lower risk, economically privileged GBM (McClelland 2019). Given public health’s history of perpetuating fear among GBM over HIV, it is not surprising that some GBM have used PrEP as a way to manage HIV-related anxieties (Gaspar et al. 2019a). Yet, the use of antiretrovirals as anti-anxiety medication has to be understood within a global context where some people living with HIV (especially people from the Global South) are still unable to access medication and healthcare for their HIV (McClelland 2019). This raises critical questions about privilege and the biopolitics of anxiety—whose health-based worries are considered alleviable and whose are not.

In Canada, another potential for over-medicalization can be observed in a strong reliance on the HIV Incidence Risk Index for Men who have Sex with Men (HIRI) to determine PrEP eligibility (Smith et al. 2012). The HIRI is a survey (e.g. age, STI history, receptive intercourse) with each response garnering specific points. The final accumulated score indicates the degree of risk a GBM is at for contracting HIV. In Canada, PrEP is generally recommended for GBM with a HIRI of 10 or higher. The use of the HIRI makes it difficult for many sexually active GBM to not be considered eligible for PrEP. For instance, based on the HIRI, a 28-year-old GBM who is a receptive anal sex partner in a monogamous relationship will be deemed a good PrEP candidate. While it is generally understood that the HIRI is an imperfect metric that needs to be coupled with counselling, its consistent use as *the* baseline tool in Canada demonstrates an implicit preference towards recommending PrEP to sexually active GBM than not. O’Byrne et al. (2019) argue that it is not a neutral act for a medical professional to suggest PrEP to a client, as this can be considered to be a form of social regulation and disciplinary power. Instruments like the HIRI and a general enthusiasm for recommending PrEP (whether or not it is actually warranted for an individual GBM) has made it so that the decision to *not* go on PrEP is now an active choice.

Moreover, in the context of a historically underfunded mental healthcare system in Canada, especially for sexual minorities (Gaspar et al. 2019b), it does become easier for clinicians to prescribe PrEP to GBM than to create therapeutic relationships that would help move some GBM towards healthier attachments to sex or drugs by attending to underlying trauma or socioeconomic precarity. PrEP does not address the factors that make an individual vulnerable to HIV and/or that reduce his mental well-being in the first place. While there is no expectation that PrEP should be able to alleviate such vulnerabilities, it is important to highlight that “removing HIV from the equation” with PrEP does not minimize the range of care GBM need to address underlying trauma (Black et al. 2020).

The potential over-medicalization through PrEP also raises issues about side effects. PrEP can affect bone density as well as liver and kidney functioning (Calabrese et al. 2016). Though these are generally considered reversible, little is known about the longer-term effects of being on PrEP. Such harms are often understood as being less consequential than HIV seroconversion. This returns us to the ambivalent nature of the *pharmakon*, where the Western ontological focus on primary pharmaceutical effects (in this case, HIV prevention) renders bodily harms as “secondary” (Perrson 2004).

The other main “side-effect” debate is risk compensation and its effect on STI rates (Calabrese et al. 2016). Though GBM on PrEP are ideally supposed to use condoms, the effectiveness of PrEP as a prophylactic does lead many GBM to forgo them. This has generated concerns over STIs, which were increasing among GBM in Canada before PrEP (Public Health Agency of Canada 2017). However, PrEP’s advent has generated an ambivalent epidemiological narrative regarding the significance of STIs. Prior to PrEP implementation, the rise in STI incidence among GBM was seen to be an exceptionally important problem, not least because of antibiotic resistant strains, but also because of the likelihood of HIV transmission and more serious morbidities among people living with HIV. However, with the desire to promote PrEP as the solution to solving the HIV epidemic, the narrative has been challenged by some to suggest that STIs may be a more innocuous concern, an inevitable by-product of PrEP use that has to be accepted and managed through routine testing. This has generated strong ambivalence in the field, with some health practitioners and policy makers remaining highly concerned, and others undisturbed by the threats of risk compensation (Holt et al. 2019).

## Sexual politics

PrEP implementation has also produced ambivalent reactions towards the cultural and political significance of condomless anal sex or barebacking. Prior to PrEP (as well as “U = U” and TasP), GBM who engaged in “unprotected anal sex” were regularly positioned in the epidemiological literature as problems to be solved (Dowsett 2009). Conversely, queer theorists framed barebacking as a form of radical resistance to public health governmentality and heteronormative society (Dean 2015; Dowsett 2017). Hence, before PrEP’s implementation, there was a clear epistemological and moral/political divide between those who considered barebacking a serious public health problem to be rectified, and those who understood it as a human response to living through a public health crisis. By changing the risks associated with forgoing condoms, PrEP has ostensibly challenged this division. Condomless anal sex has shifted from indisputably dangerous to, with PrEP, (mostly) safe.

At the community level, there have been contentious debates globally among GBM about PrEP use (Race 2016). Preliminary attitudes disparaging early adopters of PrEP as “Truvada Whores” and framing PrEP as a “party drug” demonstrate that PrEP was initially associated with “promiscuity”, “deviancy”, and an implicit resistance to “responsible behaviour” (i.e. always use condoms, limit sexual partners) (Grace et al. 2018; Knight et al. 2016). The early adoption of PrEP—especially when one had to get a prescription off-label and/or join semi-permissible grassroots buyer clubs—contained vestiges of queer rebellion, an obvious resistance to public health regulations. Thus, after Health Canada approved PrEP, for a brief moment the objectives of public health to expand PrEP access could be seen as compatible with a queer sexual politics that understands condomless anal sex as the avenue towards sexual liberation.

However, the notion of the GBM who “barebacks” with PrEP went from being a moment in counter-culture to a mainstream public health event (Dean 2015). With more GBM deciding to go on PrEP, advertising their PrEP use on social media and framing it as *the* healthy and *the* responsible choice, the counter-cultural dimensions of PrEP use have become tenuous. To have sex on PrEP is now no longer counter to mainstream public health dogma. It *is* public health dogma. Rather than being shamed for being highly sexually active, GBM may be shamed for *not* being on PrEP.

As Dossett (2017) argues, biomedical advances in HIV like PrEP have turned gay men or “men who have sex with men (MSM)” from erotic subjects into consumers: “We are no longer gay; we are men who consume sex with men. The term ‘MSM’ finally makes sense as a market niche” (p. 945). Trachman and Girard (2018) have observed that early epidemiological discourse on PrEP often reified notions of the reckless GBM who is incapable of protecting himself from HIV without a biomedical fix, rather than “highlighting the exemplary nature of gay men who protect themselves” (p. S13). Thus, PrEP’s success can overshadow decades of GBM safer sex cultures and community work done to prevent HIV (Dowsett 2009; Kippax et al. 2013), and in so doing, it can also minimize how mainstream epidemiology directly fostered homophobia and HIV stigma by routinely problematizing GBM’s sexual behaviour (Gaspar 2019).

To Race (2016), PrEP’s role in moving us towards an “AIDS free generation” evacuates the queer potentials previously associated with confronting the “abjection of HIV/AIDS”. Similarly, Dean (2015) avers that the further medicalization of sexuality through PrEP continues to wed gay politics to a politics of respectability. These critiques invoke Foucault’s warning that “we must not think that by saying yes to sex, one says no to power” (1978/1990, p. 157). While PrEP offers opportunities for GBM to minimize HIV risk, biopolitically it also subjugates, disciplines, and surveils. Assuaged by the fact that gay sex has finally been saved, it may become more difficult for some to question dominant modes of power. This is perhaps why PrEP “advocacy” has taken on a far more mainstream public health implementation science approach in Canada, while the critical structural HIV work (i.e. HIV criminalization, harm reduction programming, combating the over-policing of working class and racialized communities, and decolonizing activism) are so often sidelined in GBM health forums in Canada.

## Ambivalence, reflexivity, and the embodied researcher: conclusions

Novel biomedical technologies always arrive with uncertainty, thus setting the stage for ambivalent reactions. This is not solely due to medicine’s uncertain effects on the body, but also to its speculative impact on the body politic. Pills are not just the accumulation of chemical ingredients, but are the products of convoluted biopolitical forces (Perrson 2004). The pharmakon’s dual nature as remedy/toxin reminds us that within medicine’s capacity to save lives remains its ability to poison the (social) body. To question medicine’s shortcomings is not to diminish its positive attributes, but is rather to ask for a more comprehensive view of what biopolitical futures are possible (Rosengarten and Murphy 2019).

Despite ambivalence being a core characteristic of emergent medicines, feelings of cynicism or indifference to a novel biomedical technology can be seen as barriers to a new drug’s implementation. For social scientists, being sceptical does not automatically mean being ambivalent, as debating the truthfulness of emerging evidence is necessary for the advancement of scientific knowledge (Merton 1942/1973). However, scepticism potentially becomes problematic if it turns into feelings of cynicism or pessimism regarding the *norms* guiding a scientific field. This is not simply about debating the veracity of facts, but is instead about confronting the conflicting values and power structures embedded within the enterprise of scientific knowledge production itself. This cynicism can lead to ambivalence when it is held in contrast to the enthusiastic emotional habitus structuring PrEP implementation discourse and the social and professional pressures to perform this enthusiasm.

In order to implement a biomedical technology and get the public on board, experts must demonstrate collective confidence and certainty, not cynicism and doubt. Nonetheless, while ambivalence may make decision-making and knowledge production challenging, it paradoxically has productive capacities, with an ability to “increase reflexivity and give rise to forms of agency that defy narrow decision-making frames” (Marent et al. 2018, p. 134). To prioritize ambivalence as a site of critical reflexivity acknowledges that researchers are not just vessels that objectively make and communicate science, but they are themselves embodied subjects who are emotionally affected by and emotionally invested in their work.

With attention to the reflexive capacities of ambivalence, in this article we have examined three interrelated areas of debate regarding PrEP implementation for GBM. The first concerns epistemology and praxis. On the one hand, social scientists can acknowledge the benefits of PrEP for GBM health. On the other hand, PrEP adds to a growing chorus of biomedical triumphalism, fuelled as well by “U = U” and TasP, that can drown out considerations of the social and structural dimensions fuelling the epidemic. For social scientists, celebrating PrEP enthusiastically can sometimes feel like celebrating one’s irrelevance in the field. This is even more the case when coupled with the field’s growing enthusiasm for promoting “U = U” (Grace et al. 2020), a biomedical moment and discursive strategy that also neatly obfuscates the same field’s long history of pathologizing GBM sex through notions of “treatment optimism” and undetectable viral load (Gaspar 2019). Finally, critical social scientists may come to feel ambivalent about their paradoxical role in these epistemological and biopolitical systems, simultaneously critiquing biomedical triumphalism while routinely benefiting professionally from the advancement of (and opportunities to then critique) said biomedical triumphalism.

The second area of debate refers to clinical and epidemiological outcomes. On the one hand, PrEP can reduce vulnerabilities to HIV. On the other hand, access to PrEP in Canada favours those with socioeconomic advantage and can thus increase HIV-related inequities between highly affected groups. Conversely, a concern with under-prescribing PrEP is matched by a caution with “over-prescribing” PrEP, giving it to anxious men whose sexual practices may not necessarily warrant it, with implications on long-term side effects and the potentials for risk compensation. Of the latter, increases in STIs may or may not be terribly concerning, representing a substantial change in tone in the HIV field.

And finally, PrEP has raised issues regarding sexual politics. While PrEP may be envisioned as a radical tool that allows for increased intimacies and pleasures, conversely, it can be an additional avenue for sexuality to become increasingly medicalized, commercialized, and surveilled, another avenue to expunge dissent. Though many public health experts are now *somewhat* more accepting of condomless anal sex among GBM because of PrEP along with “U = U” and TasP, this newfound acceptance can excuse the ways in which epidemiology has problematized the lives of GBM for decades, advancing the very HIV stigma and fears that have now made PrEP such an attractive option for many anxious-ridden GBM (Gaspar 2019; Gaspar et al. 2019a).

These areas of debate represent contradictory feelings and opinions about the biopolitical norms PrEP implementation reifies. How does PrEP foster the lives of some groups while disallowing/ignoring others? How does PrEP increase a reliance on some forms of expertise, professional development, and knowledge production, while minimizing other contributions better suited at addressing health inequities? How does PrEP operate to increase forms of regulatory power and surveillance in people’s sex lives? And, with this ambivalence in mind, how are the scientists who are asking these critical questions able to do so without jeopardizing the necessary scale-up of PrEP? To implement PrEP effectively seemingly requires critical sociological questions to be put aside as “interesting caveats” in the service of the “real” work of expanding access. Yet, putting these “caveats” aside *is* a biopolitical process that will always leave these (and other) questions unanswered at the expense of what current power structures deem strategic.

As McClelland (2019) observes, strong interests in PrEP have made it difficult to assess what the *meaning* of PrEP might be. Paradoxically, to even question PrEP’s shortcomings can be framed as being anti-gay or sex-negative, a challenging allegation for those of us dedicated to addressing homophobia and HIV stigma. Relatedly, Race (2016) has documented how a “strategic optimism” to the “end of HIV/AIDS” rooted in biomedical advances like PrEP has become *compulsory* for those working in the field. Feelings of agnosticism or annoyance are considered distractions from the certainty and unbridled excitement one should be feeling about PrEP and “U = U”. Gould’s (2009) sociological work on ambivalence is useful here. Though individual scientists and health practitioners may have conflicting feelings about PrEP, the current *emotional habitus* of the HIV field requires reducing the expression of concern in public forums for the sake of a shared vision of what progress should look like—that is, scaling up PrEP. A sociological focus on emotional habitus is not an analytic distraction from addressing the structural components determining the epistemological and biopolitical systems at the centre of our critique. Rather, by emphasizing the role of ambivalence in the PrEP triumphant landscape, we are highlighting how the material and ideological forces shaping HIV professional networks come to delimit when and how as critical social scientists we are able to intervene on the root causes of health inequities.

Indeed, giving voice to ambivalence protects the integrity of science by protecting the integrity of the scientist as an embodied, material subject. Marxists have long averred that alienation can be detrimental to one’s well-being (Yuill 2005). While this alienation is largely a question of owning the means of production, Hochschild’s (1983/2012) seminal work on emotion management in the workplace has shown that continued efforts to control one’s “negative” feelings can lead to fatigue and indifference (i.e. burnout), and is ultimately detrimental to a sense of self. Continually displacing cynicism or doubt in the service of collaboration may seem necessary to advance a specific implementation goal, but this emotion management work comes at the potential cost of alienation. This alienation, when left unacknowledged, effectively takes energy from those social scientists responsible for considering the important pressing question: *what’s next?* However, attending to ambivalence among researchers recognizes the embodied nature of the scientist who must make concessions for their craft. While we can only speculate here, more empirical work should be done to examine the extent of potential alienation in the HIV sector (itself evolving quickly under biomedical triumphalism), its myriad sources, and its effect on service delivery and community mobilization efforts. Recognizing the emotional foundations of HIV work—including the labour of researchers and health practitioners operating in increasingly competitive professional environments—need not be considered a distraction from “real” science, but may actually be the site to creatively develop novel and pertinent lines of inquiry.

Our analysis does not exclusively consider the subjectivity of GBM negotiating sexual risk in their everyday lives, but engages with the subjectivity of the HIV researcher actively working to better understand and respond to the complexity of biomedical advances. Such a move can seem like a grave error in the public health and biomedical sciences, where a Western predisposition to the ideals of positivism and evidence-based argumentation demands the removal of the researching subject—and certainly any contradictory feelings they may carry—out of the equation. Emotions are not science. One can debate arguments rationally, but is not to confess that some scientific debates lead to ambivalence.

However, while science is not *supposed* to be personal, emotional, or political, it very much is these things. Accounting for this messiness, and in particular, the mixed feelings that may emerge due to the movement of scientific fields like GBM health research and HIV, can, we believe, extend beyond a simple evaluation of “objective facts” towards a deeper ethical reflection on the biopolitical significance of our work and what future we are striving for.
